# Complete genomes of clade A1 and B *Enterococcus faecium* isolates harboring pHTβ, a *vanA*-type vancomycin-resistant pMG1-like plasmid

**DOI:** 10.1128/mra.00684-25

**Published:** 2025-09-26

**Authors:** Jun Kurushima, Takahiro Nomura, Natsuko Ota, Haruyoshi Tomita

**Affiliations:** 1 Laboratory of Bacterial Drug Resistance, Gunma University, Graduate School of Medicine 12925, Maebashi, Japan; 2 Department of Bacteriology, Gunma University, Graduate School of Medicine 12925, Maebashi, Japan; Loyola University Chicago, Chicago, Illinois, USA

**Keywords:** *Enterococcus faecium*, plasmid, horizontal gene transfer, VRE, vancomycin, conjugation, AMR, antimicrobial resistance

## Abstract

Enterococcal conjugative plasmids play a key role in the widespread expansion of antimicrobial resistance genes among *Enterococcus faecium* via horizontal gene transfer. Herein, we report the complete genomes of clinical *E. faecium* isolates harboring pHTβ, a pMG1-like plasmid containing an inserted vancomycin resistance operon.

## ANNOUNCEMENT

Vancomycin-resistant *Enterococcus faecium* (VRE) is one of the most concerning antimicrobial-resistant (AMR) bacterial pathogens (1). In *E. faecium*, AMR genes are acquired through horizontal gene transfer mediated by conjugative plasmids (2). Plasmids within the pMG1-like group often carry AMR genes and possess a high capacity for efficient conjugative transfer (3, 4). pHTβ, a pMG1-like plasmid that carries the *vanA*-type vancomycin resistance operon, has been used as an experimental model for studying pMG1-like plasmid biology (5). Here, we report the complete genome sequences of two clinical VRE strains pHTβ plasmid, MEM18094 and OEK20567, which were isolated from feces of inpatients of the university hospital in Japan (6).

The bacterial strains were cultured in brain heart infusion at 37°C overnight. Pelleted cell suspensions were lysed with 120 µL of TE buffer containing lysozyme (MPBio), metapolyzyme (Sigma-Aldrich), and RNase A (ITW Reagents). This mixture was incubated at 37°C for 25 min, followed by a 5 min incubation at 65°C with Proteinase K (VWR Chemicals) at a final concentration of 0.1 mg/mL and 0.5% sodium dodecyl sulfate (Sigma-Aldrich). Genomic DNA was purified using an equal volume of solid-phase reversible immobilization beads and resuspended in elution buffer (10 mM Tris-HCl, pH 8.0). The obtained DNA was quantified using the Quant-iT dsDNA HS (ThermoFisher Scientific) assay on an AF2200 plate reader (Eppendorf Ltd).

For short-read sequencing, genomic DNA libraries were prepared using the Nextera XT Library Prep Kit (Illumina) and sequenced on a NovaSeq 6000 sequencer (Illumina) following a 250-bp paired-end protocol. Reads were adapter-trimmed using Trimmomatic version 0.30 (7) with a sliding window quality cutoff of Q15. The obtained raw read numbers of these short reads were 2,207,968 and 1,346,804 for MEM18094 and OEK20567, respectively. For long-read sequencing, DNA libraries were prepared with the SQK-RBK114.96 kit (Oxford Nanopore Technologies), using 200–400 ng of high-molecular-weight DNA. Barcoded samples were pooled into a single sequencing library and loaded onto a FLO-MIN114 (R.10.4.1) flow cell in a GridION (Oxford Nanopore Technologies). The obtained raw read numbers of these long reads were 61,556 and 52,073 for MEM18094 and OEK20567, respectively. The N50 scores of the long reads were 7,780 and 10,231 for MEM18094 and OEK20567, respectively. Hybrid assembly was performed using Unicycler version 0.4.0 with default parameters (8). Read coverages were 164× and 109× for MEM18094 and OEK20567 genomes, respectively. Resulting contigs were then annotated using the DDBJ Fast Annotation and Submission Tool (DFAST) v.1.3.6 pipeline (9).

Basic statistics of the genome assemblies are presented in Table 1. MEM18094 and OEK20567 have seven and two plasmids, respectively. MEM18094 belongs to clonal complex 17 (CC17), a representative of Clade A1, a hospital-associated *E. faecium* lineage with the highest VRE infection risk (10). OEK20567 could not be assigned to a known sequence type (ST); however, it is closely related to ST695, ST855, and ST2466 within CC94. This clonal complex belongs to Clade B, a community-associated *E. faecium* lineage with low VRE infection risk. Notably, despite the distinct genomic lineage backgrounds of their hosts, the pHTβ plasmids from both genomes were completely identical, exhibiting no single-nucleotide variants.

**TABLE 1:** Statistics of the genome assemblies

Strain	Contig	Length (bp)	Type	Topology	Accession number
MEM18094	Chromosome	2,915,325	Chromosome	Circular	AP041217.1
	pMEM18094a	277,387	Plasmid	Circular	AP041218.1
	pHTbeta	63,809	Plasmid	Circular	AP041219.1
	pMEM18094c	10,645	Plasmid	Circular	AP041220.1
	pMEM18094d	6,189	Plasmid	Circular	AP041221.1
	pMEM18094e	4,384	Plasmid	Circular	AP041222.1
	pMEM18094f	1,735	Unplaced contig	Linear	AP041223.1
	pMEM18094g	831	Unplaced contig	Linear	AP041224.1
OEK20567	Chromosome	2,700,242	Chromosome	Circular	AP041225.1
	pOEK20567a	175,595	Plasmid	Circular	AP041226.1
	pHTbeta	63,809	Plasmid	Circular	AP041227.1
	pOEK20567c	4,384	Plasmid	Circular	AP041228.1

## Data Availability

The assembled genomic data from this study were deposited in the DNA Data Bank of Japan under BioProject accession number PRJDB20792. BioSample accession numbers for the strains MEM18094 and OEK20567 are SAMD00916348 and SAMD00916349, respectively. The raw sequence reads were deposited in the Sequence Read Archive with accession number DRA021326.
